# Retraction Note: α2,6-Sialylation mediates hepatocellular carcinoma growth in vitro and in vivo by targeting the Wnt/β-catenin pathway

**DOI:** 10.1038/s41389-020-0211-6

**Published:** 2020-02-27

**Authors:** Y. Zhao, A. Wei, H. Zhang, X. Chen, L. Wang, H. Zhang, X. Yu, Q. Yuan, J. Zhang, S. Wang

**Affiliations:** 1 Department of Biochemistry and Molecular Biology, Institute of Glycobiology, Dalian Medical University, Dalianm, Liaoning Province China; 2 School of Life Science and Medicine, Dalian University of Technology, Dalianm, Liaoning Province China; 3 Department of Pathology, Dalian Medical University, Dalianm, Liaoning Province China

**Keywords:** Glycobiology, Liver cancer

**Retraction of: Oncogenesis**

10.1038/oncsis.2017.40 published online 29 May 2017

The authors have retracted this article [1] because there are errors in some images. In Fig. 2g, a colony picture for Mock group was incorrect. In Fig. 2k, the invasion pictures for Mock and overexpression ST6Gal-1 cells were incorrect. In Fig. 3g, the colony picture for shNC was incorrect. In Fig. 4f, the authors mistakenly provided WB band for GAPDH. In Fig. 4g, the IHC pictures for 10w in Negative Control and DENA-induced Groups were incorrect, and in Fig. 7f, a IHC picture for GSK-3β in DENA-induced Group (10w) was presented incorrectly. Due to these errors the findings are no longer reliable.

The following authors have agreed to this retraction - Zhao Y, Chen X, Wang L, Zhang J, Wang S, Wei A.

The following authors have not responded to any correspondence from the publisher about this retraction - Zhang H, Zhang H, Yu X, Yuan Q.

[1] Zhao Y, Wei A, Zhang H, Chen X, Wang L, Zhang H, Yu X, Yuan Q, Zhang J, Wang S. α2, 6-Sialylation mediates hepatocellular carcinoma growth in vitro and in vivo by targeting the Wnt/β-catenin pathway. *Oncogenesis* **6**, e343. 10.1038/oncsis.2017.40 (2017).

